# A Validated Stability-Indicating RP-UPLC Method for Simultaneous Determination of Desloratadine and Sodium Benzoate in Oral Liquid Pharmaceutical Formulations

**DOI:** 10.3797/scipharm.1111-08

**Published:** 2011-12-12

**Authors:** Navneet Kumar, Dhanaraj Sangeetha, Pingili Sunil Reddy, Lakkireddy Prakash

**Affiliations:** 1 Analytical Research and Development, Integrated Product Development, Dr. Reddy's Laboratories Ltd., Bachupally, Hyderabad-500072, A.P., India; 2 Department of Chemistry, S.A.S., V.I.T. University, Vellore-632014, Tamilnadu, India

**Keywords:** Development, Validation, Degradation, Stability-indicating, UPLC-UV, Desloratadine, Sodium benzoate

## Abstract

A novel, sensitive and selective stability-indicating gradient reverse phase ultra performance liquid chromatographic method was developed and validated for the quantitative determination of desloratadine and sodium benzoate in pharmaceutical oral liquid formulation. The chromatographic separation was achieved on Acquity BEH C8 (100 mm × 2.1 mm) 1.7 μm column by using mobile phase containing a gradient mixture of solvent A (0.05 M KH_2_PO_4_ and 0.07 M triethylamine, pH 3.0) and B (50:25:25 v/v/v mixture of acetonitrile, methanol and water) at flow rate of 0.4 mL/min. Column temperature was maintained at 40°C and detection was carried out at a wavelength of 272 nm. The described method shows excellent linearity over a range of 0.254 μg/mL to 76.194 μg/mL for desloratadine and 1.006 μg/mL to 301.67 μg/mL for sodium benzoate. The correlation coefficient for desloratadine and sodium benzoate was more than 0.999. To establish stability-indicating capability of the method, drug product was subjected to the stress conditions of acid, base, oxidative, hydrolytic, thermal and photolytic degradation. The degradation products were well resolved from desloratadine and sodium benzoate. The developed method was validated as per international ICH guidelines with respect to specificity, linearity, LOD, LOQ, accuracy, precision and robustness.

## Introduction

**Fig. 1:**
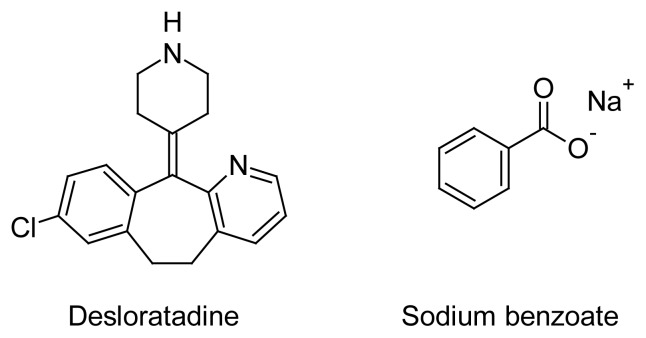
Chemical structures of desloratadine and sodium benzoate.

Desloratadine, 8-chloro-11-(piperidin-4-ylidene)-6,11-dihydro-5*H*-benzo[5,6]cyclohepta-[1,2-*b*]pyridine (Fig. 1), is a peripheral histamine H1-receptor anatagonist commonly used to treat allergy symptoms such as nasal and nonnasal symptoms of seasonal allergic rhinits. Desloratadine is available as 5 mg tablets and 0.5 mg/mL syrup [1, 2]. Compared to tablet and capsule, liquid formulations favour a most rapid absorption of active substance. Liquid preparations are particularly susceptible to mocrobial growth because of the nature of their ingradtient. Such preparations require the presence of preservatives and antimicrobial agents to prevent chemical alteration and degradation of drug substance.

Sodium benzoate, sodium salt of benzoic acid, is widely used as antimicrobial preservatives in cosmetics, foods and pharmaceutical products. Sodium benzoate used at a concentrataion of 0.02–0.5% in oral medicines, 0.5% in parenteral products and 0.1–0.5% in cosmetics. Sodium benzoate (Fig. 1) has been used for the preservation of desloratadine syrup. The analysis of preservatives in commercial pharmaceutical products is particularly important for both quality assurance and consumer protection. International conference on harmonization and USFDA guidance recommends that the finished product shelf-life specification should also include identification test, preservative content and limits for antimicrobial preservative present [3, 4]. Therefore, the analysis of desloratadine into oral syrup in combination with preservative is required and essential. Some liquid chromatography (LC) methods are available for the determination of desloratadine in biological fluids [5–8]. Literature reported few RP-LC methods for the quantification desloratadine in pharmaceutical dosage forms [9–11]. Spectrofluorimetric method was also reported for the determination of desloratadine in dosage forms [12]. No pharmacopoeial monograph is available for desloratadine syrup. Several analytical procedures have been reported for the determination of sodium benzoate seperately or in combination with the other drugs by HPLC [13–19]. These methods may not be suitable for simultaneous determination of desloratadine and sodium benzoate together in one chromatographic run.

To the best of our knowledge, there is no stability-indicating LC method reported for the simultaneous estimation of desloratadine and sodium benzoate in oral liquid formulation. Therefore, attempts were made in this study to develop a fast, sensitive, selective and stability-indicating reverse phase ultra-performance liquid chromatography (UPLC) method for the simultaneous determination of desloratadine and sodium benzoate in oral liquid formulation. The proposed method is able to seperate desloratadine and sodium benzoate with each other and from its impurities, degradation products and placebo components. The developed LC method was validated with respect to specificity, linearity, limit of detection and quantification, precision, accuracy and robustness. Force degradation studies were performed on the placebo and drug product. Developed method separates all degradation products from desloratadine and sodium benzoate and exhibits stability-indicating nature. These studies were performed in accordance with established International Conference on Harmonization (ICH) guidelines.

## Results and Discussion

### Method Development and Optimization

The main objective of the chromatographic method was to separate and quantitate desloratadine and sodium benzoate in presence of degradation products and other placebo components like flavor agents and colors. An isocratic method was employed using 0.05 M potassium dihydrogen *ortho*-phosphate (pH 3.5) and acetonitrile in the ratio of 75:25 as mobile phase, Acquity BEH C18 (100 mm × 2.1 mm) 1.7 μm column with flow rate of 0.4 mL/min on UPLC equipped with photo diode array detector. Sodium benzoate peak was eluted along with placebo peaks and base degradant eluted too late. To separate sodium benzoate from placebo peaks and reduce the run time an attempt was made with gradient elution with mobile phase 0.05 M potassium dihydrogen *ortho*-phosphate buffer (pH 3.5) as solvent-A and solvent-B (mixture of acetonitrile and water in the ratio of 80:20 v/v). Sodium benzoate was resolved from placebo peaks but desloratadine peak eluted late along with degradation product. To resolve the desloratadine from degradation product and reduce the run time, pH of solvent A changed to 3.0 and solvent B modified to the mixture of acetonitrile, methanol and water in the ratio of 50:25:25 v/v/v, respectively and Acquity BEH C8 (100 mm × 2.1 mm) 1.7 μm column was selected for separation. On the optimization of gradient program, desloratadine and sodium benzoate peaks were well resolved from degradation products but peak tailing was observed more than 1.8. To reduce the peak tailing solvent-A was modified to 0.05 M KH_2_PO_4_ and 0.07 M triethylamine, adjusted pH to 3.0 with *ortho*-phosphoric acid and results to peak tailing less than 1.2. Based on these experiments, the final optimized conditions are described below.

Acquity BEH C8 (100 mm × 2.1 mm) 1.7 μm was used as the stationary phase. The mobile phase A consisted of 0.05 M KH_2_PO_4_ and 0.07 M triethylamine, adjusted pH 3.0 with *ortho*-phosphoric acid and mobile phase B contained a mixture of acetonitrile, methanol and water in the ratio of 50:25:25 v/v/v, respectively. The flow rate was 0.4 mL/min with a gradient program of (time (min)/%B) 0/27, 4.5/32.4, 5.2/80, 5.4/80, 5.5/27 and 7/27. The column temperature was maintained at 40°C and detection was monitored at 272 nm. The injection volume was 2 μL. The typical retention time of sodium benzoate and desloratadine was about 2.51 and 4.51 min, respectively.

### Validation of the Method

The proposed method was validated by determining its performance characteristics regarding specificity, accuracy, precision, limit of detection and quantification, linearity, range and robustness [4, 20, 21].

### System suitability

System suitability shall be checked for the conformance of suitability and reproducibility of chromatographic system for analysis. System suitability was determined before sample analysis from five replicate injections of the standard solution containing 50 μg/mL of desloratadine and 200 μg/mL of sodium benzoate. The acceptance criteria were less than 2% relative standard deviation (RSD) for peak areas, USP tailing factor less than 2.0 and USP plate count more than 5000 for desloratadine and sodium benzoate peaks from standard solution. All critical parameters tested met the acceptance criteria (Table 1).

### Specificity

**Fig. 2:**
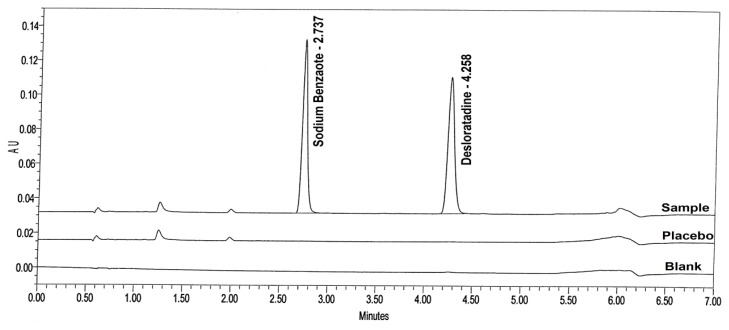
Overlay chromatogram of blank, placebo and sample.

Specificity is the ability of the method to measure the analyte response in the presence of its potential degradants and placebo matrix. In the present study, injections of blank and placebo were performed to demonstrate the interference with the elution of desloratadine and sodium benzoate. These results demonstrate that there was no interference at the retention time of desloratadine and sodium benzoate from the other compounds and, therefore, confirms the specificity of the method (Fig. 2).

### Forced degradation studies

Force degradation studies of drug product were also performed to evaluate the stability-indicating property and specificity of proposed method. Stress studies were performed at the concentration of 50 μg/mL of desloratadine and 200 μg/mL of sodium benzaoate on syrup formulation. Peak purity test was carried out for the desloratadine and sodium benzoate peaks by using PDA detector on stress samples. All the solutions used in forced degradation studies were prepared by dissolving the drug product in small volume of stressing agents. After degradation, these solutions were diluted with diluent to yield stated desloratadine and sodium benzoate concentration of about 50 μg/mL and 200 μg/mL, respectively. Conditions employed for performing the stress studies were as follows [4, 20, 21]:

#### Acid induced degradation

**Fig. 3:**
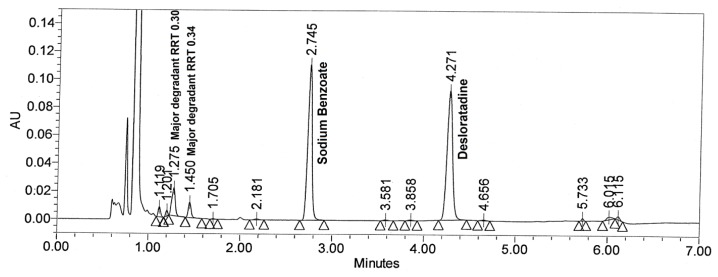
Typical chromatogram of acid degraded drug product

Acid hydrolysis was performed in 0.1 N HCl at 50°C for 20 hr but no degradation was observed. To achieve degradation, drug product was treated with 1 N HCl at 60°C for 20 hr. Fig 3 shows major degradation peaks at RRT 0.30 and 0.34 with respect to desloratadine. All the major and minor degradation products were well separated from desloratadine and sodium benzoate peaks. The peak purity was checked for both analytes and the results are summarized in Table 2.

#### Base induced degradation

**Fig. 4:**
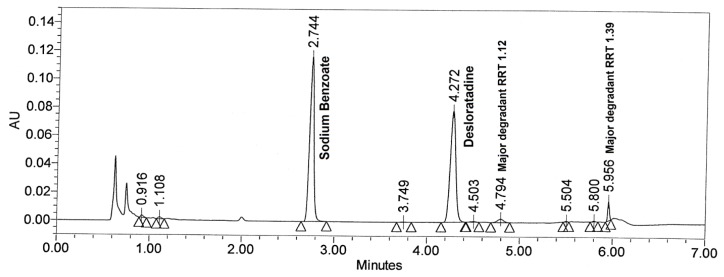
Typical chromatogram of base degraded drug product

Base hydrolysis was carried out in 0.1 N NaOH at 50°C for 20 hr, but degradation of desloratadine was found to be less than 2% and sodium benzoate found stable. To increase the degradation, drug product was subjected to base hydrolysis in 1 N NaOH at 60°C for 20 hr. Fig 4 shows that the major degradation peaks found at RRT 1.12 and 1.39 with respect to desloratadine. All the major and minor degradation products were well separated from desloratadine and sodium benzoate peaks. The peak purity was checked for both analytes and the results are summarized in Table 2.

#### Water induced degradation

Water hydrolysis was performed in purified water at 60°C for 20 hr. Desloratadine and sodium benzoate were found stable under water hydrolysis stress conditions. The peak purity was checked for both analytes and the results are summarized in Table 2.

#### Hydrogen peroxide induced degradation

**Fig. 5:**
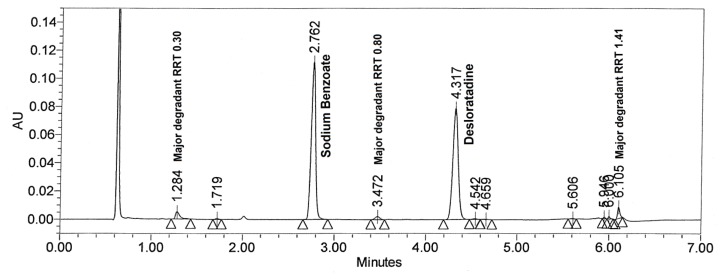
Typical chromatogram of peroxide degraded drug product

Oxidative degradation was performed in 1% hydrogen peroxide at 50°C for 20 hr but degradation of desloratadine and sodium benzoate were found to be less than 2% and 0.4%, respectively. To achieve optimum degradation, drug product was treated with 6% hydrogen peroxide at 60°C for 20 hr. Fig 5 shows that the major degradation peaks found at RRT 0.30, 0.80 and 1.41 with respect to desloratadine. All the major and minor degradation products were well separated from desloratadine and sodium benzoate peaks. The peak purity was checked for both analytes and the results are summarized in Table 2.

#### Heat induced degradation

The drug product was exposed to dry heat at 105°C for 24 hr. Following removal from the oven, sample was prepared for analysis as previously described under sample preparation. Desloratadine and sodium benzoate were found stable under thermal stress conditions. The peak purity was checked for both analytes and the results are summarized in Table 2.

#### Photo-degradation

Photo degradation studies were carried out according to option 2 of ICH guideline Q1B [20]. Samples were exposed to white florescent light for an overall illumination of 1.2 million lux hours and near UV radiation with an overall illumination of 200 watt/m^2^/hr at 25°C. Following removal from the photo-stability chamber, sample was prepared for analysis as previously described under sample preparation. No degradation was observed during photolytic degradation condition. The peak purity was checked for desloratadine and sodium benzoate and the results are summarized in Table 2.

### Limits of Detection (LOD) and Quantification (LOQ)

The LOD and LOQ were determined at a signal-to-noise ratio of 3:1 and 10:1, respectively, by injecting a series of dilute solutions with known concentrations. The limit of detection and limit of quantification values of desloratadine and sodium benzoate are reported in Table 3.

### Linearity

Linearity test solutions were prepared at seven concentrations ranging from LOQ to 150% levels of test concentration (LOQ – 76.2 μg/mL for desloratadine and LOQ – 150.8 μg/mL for sodium benzoate). The peak area was plotted against the concentration at each level and a calibration curve was generated by least-squares linear regression analysis. The coefficient correlation, slope, y-intercept of the calibration curve and % bias at 100% response are reported (Table 3) and results show that an excellent correlation existed between peak area and concentration of desloratadine and sodium benzoate.

### Precision

The precision of method was verified by repeatability and intermediate precision. Repeatability was checked by injecting six individual preparations of desloratadine syrup containing desloratadine and sodium benzoate at 10%, 50%, 100% and 150% level of test concentration (5.1, 25.4, 50.8, and 76.2 μg/mL for desloratadine; and 20.1, 100.6, 201.1 and 301.7 μg/mL for sodium benzoate). The intermediate precision of the method was also evaluated using different analyst and different instrument and performing the analysis on different days. Intermediate precision was determined by injecting six individual preparations of desloratadine syrup containing desloratadine and sodium benzoate at 10%, 50%, 100% and 150% level of test concentration (5.1, 25.4, 50.8, and 76.2 μg/mL for desloratadine; and 20.1, 100.6, 201.1 and 301.7 μg/mL for sodium benzoate). The relative standard deviation of the areas of each peak was calculated and found to less than 0.9% in repeatability and less than 1.3% in intermediate precision, which confirms the good precision of the method. The %RSD values are presented in Table 4.

### Accuracy

Accuracy of the method for desloratadine and sodium benzoate was evaluated in triplicate at 10%, 50%, 100% and 150% level of test concentration (5.1, 25.4, 50.8, and 76.2 μg/mL for desloratadine; and 20.1, 100.6, 201.1 and 301.7 μg/mL for sodium benzoate). The percentage recoveries for both components were calculated (Table 5). The percentage mean recovery of desloratadine and sodium benzoate from the formulation varied from 98.9 to 102.0% indicating that the developed method was accurate for the determination of desloratadine and sodium benzoate in pharmaceutical formulation.

### Robustness

The robustness of the method was evaluated during development by making small, but deliberate, changes to the method parameters. The variables evaluated in the study were pH of the mobile phase buffer (± 0.2), column temperature (± 5°C), flow rate (± 0.04 mL/min) and % organic in the mobile phase (± 10%) and system suitability parameters such as % RSD, retention time, tailing factor and theoretical plates of desloratadine and sodium benzoate standard were studied. In all the deliberate varied chromatographic conditions, system suitability parameters met the acceptance criteria (Table 6). Thus, the method was found to be robust with respect to variability in applied conditions.

### Stability of analytical solutions

The solution stability of desloratadine and sodium benzoate in the assay method was investigated by leaving standard and sample solutions in tightly capped volumetric flask at room temperature for 48 hr. The same sample solutions were analyzed at the end of the study period against freshly prepared standard solutions. The variability in the assay of both substances was within ± 3% during solution stability. The results from solution stability experiments confirmed that sample solution and standard solutions were stable up to 48 hr.

## Experimental

### Reagents and chemicals

Standard and syrup of desloratadine was supplied by Dr. Reddy’s laboratories limited, Hyderabad, India. Sodium benzoate was procured from United States Pharmacopoeia (USP). The HPLC grade acetonitrile and methanol; and analytical grade potassium di-hydrogen *ortho-*phosphate, trietylamine and *ortho*-phosphoric acid were purchased from Merck, Mumbai, India. High purity water was prepared by using Millipore Milli-Q Plus water purification system (Millipore, Milford, MA, USA).

### Equipment

The chromatography analysis was performed using Waters Acquity^TM^ UPLC separation module (Waters Corporation, Milford, USA) equipped with PDA detector, binary solvent manager and auto sampler system. The output signals were monitored and processed using Empower 2 software. Cintex digital water bath was used for hydrolysis studies. Photo-stability studies were carried out in photo-stability chamber (Sanyo, Leicestershire, UK). Thermal stability studies were performed in a dry air oven (Cintex, Mumbai, India). The pH of the solutions was measured by a pH meter (Mettler-Toledo, Switzerland).

### Chromatographic Conditions

The separation was achieved on Acquity BEH C8 (100 mm × 2.1 mm) 1.7 μm column with mobile phase containing a gradient mixture of solvent A (0.05 M potassium dihydrogen *ortho*-phosphate and 0.07 M trietylamine, pH adjusted to 3.0 with *ortho*-phosphoric acid) and B (50:25:25 v/v/v mixture of acetonitrile, methanol and water) at a flow rate of 0.4 mL/min. The gradient program (Time(min)/%B) was set 0/27, 4.5/32.4, 5.2/80, 5.4/80, 5.5/27 and 7/27. The column oven temperature was maintained at 40°C and eluted compounds were monitored at the wavelength of 272 nm. The sample injection volume was 2 μL.

### Preparation of Standard Solution

Buffer (0.05 M KH_2_PO_4_, pH 3.0) and methanol in the ratio of 50:50 v/v was used as diluent. The stock solutions of desloratadine (0.25 mg/mL) and sodium benzoate (0.5 mg/mL) were prepared by dissolving an appropriate amount of analyte in diluent, separately. Working standard solution was prepared in diluent from mixing above stock solutions of desloratadine and sodium benzoate with final concentration of 50 μg/mL and 200 μg/mL, respectively.

### Preparation of Sample Solution

An amount (about 6 gm) of oral solution equivalent to 2.5 mg desloratadine was transferred into 50 mL volumetric flask, added 35 mL of diluent and ultrasonicated for 15 min, and then diluted to volume with diluent to give a solution containing 50 μg/mL of desloratadine and 200 μg/mL of sodium benzoate.

## Conclusion

A simple and efficient reversed-phase UPLC method was developed and validated for quantitative analysis of desloratadine and sodium benzoate in pharmaceutical dosage forms. The method was found to be precise, accurate, linear, robust and rugged during validation. Satisfactory results were obtained from the validation of the method. The method is stability-indicating and can be used for routine analysis of production samples and to check the stability of the desloratadine syrup.

## Figures and Tables

**Tab. 1:** System suitability results

Parameters	Acceptance criteria	Desloratadine		Sodium benzoate	

		Precision	Intermediate precision	Precision	Intermediate precision
Area (%RSD, n=5)	≤ 2.0	0.3	0.2	0.1	0.3
USP Plate count	> 5000	18316	23951	13652	16936
USP Tailing	≤ 2.0	1.2	1.0	1.2	1.1

**Tab. 2:** Summary of forced degradation results

	Desloratadine			Sodium benzoate		
	
Stress Condition	% Degradation	Purity angle	Purity threshold	% Degradation	Purity angle	Purity threshold
Acid hydrolysis (1 N HCl at 60°C, 20 hr)	2.7	0.146	0.329	3.8	0.074	0.342
Base hydrolysis (1 N NaOH at 60°C, 20 hr)	13.8	0.064	0.287	0.0	0.075	0.356
Oxidation (6% H_2_O_2_ at 60°C, 20 hr)	13.3	0.068	0.257	1.6	0.102	0.334
Thermal (At 105°C, 24 hr)	0.5	0.059	0.250	0.0	0.060	0.330
Hydrolytic (Water at 60°C, 20 hr)	1.5	0.074	0.250	0.0	0.070	0.345
Photolytic (1.2 million lux hr visible light and 200 wh/m^2^ UV light)	0.0	0.089	0.253	0.0	0.057	0.324

**Tab. 3:** Evaluation of LOD, LOQ and linearity data

Parameter	Desloratadine	Sodium benzoate
LOD (μg/mL)	0.086	0.342
LOQ (μg/mL)	0.254	1.006
Linearity range (μg/mL)	0.254–76.194	1.006–301.670
Correlation coefficient	0.9998	0.9999
Intercept (a)	−936.968	123.469
Slope (b)	7370.222	1732.966
Bias at 100% response	−0.03	0.00

**Tab. 4:** Precision results determined during method validation

Parameter	Spiked level	% RSD (n=6)	

		Desloratadine	Sodium benzoate
Repeatability	10%	0.9	0.2
	50%	0.7	0.1
	100%	0.2	0.1
	150%	0.5	0.2

Intermediate Precision	10%	0.5	0.6
	50%	1.2	1.3
	100%	0.5	0.4
	150%	0.3	0.3

**Tab. 5:** Accuracy results

Spiked level		Desloratadine	Sodium benzoate
10%	% Recovery	101.1	102.0
	% RSD	0.5	0.2

50%	% Recovery	100.7	100.5
	% RSD	1.0	0.1

100%	% Recovery	99.9	100.8
	% RSD	0.2	0.1

150%	% Recovery	98.9	99.7
	% RSD	0.7	0.1

Mean % recovery and %RSD for three determinations.

**Tab. 6:** Robustness results of UPLC method

Variation in chromatographic condition	Observed system suitability parameters							

	Sodium Benzoate				Desloratadine			

	*t_R_*^a^	*A*^b^	*T*^c^	*N*^d^	*t_R_*^a^	*A*^b^	*T*^c^	*N*^d^
Column Temperature 35°C	2.920	0.2	1.1	16726	4.311	0.4	1.1	23937
Column Temperature 45°C	2.581	0.1	1.1	16456	4.220	0.2	1.1	24810
Flow rate 0.36 mL/min	3.053	0.1	1.1	16463	4.676	0.3	1.1	25236
Flow rate 0.44 mL/min	2.493	0.2	1.1	13913	3.929	0.3	1.1	20747
Acetonitrile 90%	2.856	0.0	1.2	10864	4.580	0.1	1.1	9559
Acetonitrile 110%	2.632	0.1	1.2	8645	3.960	0.1	1.1	11869
Methanol 90%	2.722	0.0	1.2	14235	4.148	0.1	1.1	20175
Methanol 110%	2.734	0.1	1.4	9104	4.308	0.1	1.3	10681
Mobile Phase Buffer pH 2.8	2.801	0.5	1.1	16206	3.404	0.1	1.1	19877
Mobile Phase Buffer pH 3.2	2.668	0.1	1.3	9748	4.827	0.1	1.2	15093

a Retention time (min) of the analyte peak.

b % RSD of the analyte peak areas from 5 injections.

c Tailing factor of the analyte peak.

d Plate count of the analyte peak.
